# Discovery of *lahS* as a Global Regulator of Environmental Adaptation and Virulence in *Aeromonas hydrophila*

**DOI:** 10.3390/ijms19092709

**Published:** 2018-09-11

**Authors:** Yuhao Dong, Yao Wang, Jin Liu, Shuiyan Ma, Furqan Awan, Chengping Lu, Yongjie Liu

**Affiliations:** Joint International Research Laboratory of Animal Health and Food Safety, College of Veterinary Medicine, Nanjing Agricultural University, Nanjing 210095, China; 2015207019@njau.edu.cn (Y.D.); 2015107061@njau.edu.cn (Y.W.); 2017207019@njau.edu.cn (J.L.); 2017107059@njau.edu.cn (S.M.); 2015207039@njau.edu.cn (F.A.); lucp@njau.edu.cn (C.L.)

**Keywords:** *Aeromonas hydrophila*, LysR-family, *ΔlahS*, global regulator, virulence

## Abstract

*Aeromonas hydrophila* is an important aquatic microorganism that can cause fish hemorrhagic septicemia. In this study, we identified a novel LysR family transcriptional regulator (LahS) in the *A. hydrophila* Chinese epidemic strain NJ-35 from a library of 947 mutant strains. The deletion of *lahS* caused bacteria to exhibit significantly decreased hemolytic activity, motility, biofilm formation, protease production, and anti-bacterial competition ability when compared to the wild-type strain. In addition, the determination of the fifty percent lethal dose (LD_50_) in zebrafish demonstrated that the *lahS* deletion mutant (*ΔlahS*) was highly attenuated in virulence, with an approximately 200-fold increase in LD_50_ observed as compared with that of the wild-type strain. However, the *ΔlahS* strain exhibited significantly increased antioxidant activity (six-fold). Label-free quantitative proteome analysis resulted in the identification of 34 differentially expressed proteins in the *ΔlahS* strain. The differentially expressed proteins were involved in flagellum assembly, metabolism, redox reactions, and cell density induction. The data indicated that LahS might act as a global regulator to directly or indirectly regulate various biological processes in *A. hydrophila* NJ-35, contributing to a greater understanding the pathogenic mechanisms of *A. hydrophila.*

## 1. Introduction

*Aeromonas hydrophila* is a gram-negative bacterium that is widely distributed in various aquatic environments, such as rivers, lakes, and swamps. In addition, *A. hydrophila* has a diverse host range, which includes fish, birds, amphibians, reptiles, and mammals [1]. Motile aeromonas septicemia (MAS) caused by this bacterium has caused serious damage to the aquaculture industry. In recent years, this bacterium has also been confirmed as an important pathogen of various human diseases, including diarrhea, sepsis, necrotizing fasciitis, meningitis, and hemolytic uremic syndrome [2,3]. *A. hydrophila* has a variety of factors that are associated with virulence, such as exotoxins, S-layers, extracellular enzymes and secretion systems [4]. Of these factors, hemolytic molecules may contribute to the reddening skin and systemic hemorrhagic septicemia; symptoms that are observed in diseased fish infected with *A. hydrophila*, and have been defined as one of the major virulence markers of this bacterium [5].

The expression of virulence factors in response to changes in environmental conditions is especially important for pathogenic bacteria, and this process is commonly governed by a complex network of regulatory elements [6]. Histone-like nucleoid structuring protein (H-NS) has been reported as a negative regulator of hemolytic activity by acting as a repressor of hemolysin gene expression in *Vibrio anguillarum* [7]. Sigma factor RpoE controls the development of hemolytic activity and virulence in *Vibrio harveyi* [8]. A transcriptional regulator of the MarR family, Eha, is required for the bacterial infection and the transcriptional regulation of important virulence factors in *Edwardsiella tarda*, including those that are associated with hemolytic activity [9]. Recently, the LysR family of transcriptional regulators (LTTRs), which is a well-characterized group of transcriptional regulators, has been shown to be global transcriptional regulators in a variety of bacteria [10,11]. LTTRs can regulate a diverse set of genes, such as those involved in metabolism, pilus synthesis, biofilm formation, antioxidant activity, acid resistance, toxin production, and drug efflux [12,13,14,15,16,17]. More often than not, LTTRs indirectly affect the expression of virulence factors by regulating virulence-related transcriptional regulators. For example, LrhA in enterohemorrhagic *Escherichia coli* positively regulates the expression of LEE genes by the regulation of their master regulators PchA and PchB [18]. The LysR family transcription factor LeuO in *Vibrio cholerae* regulates the transcription of the CarRS two-component regulatory system to control *almEFG* expression, which contributes to cationic antimicrobial peptide (CAMP) resistance by glycosylation of lipid A [19]. In *Haemophilus parasuis*, approximately 500 differentially expressed genes were identified, resulting from the deletion of *oxyR*, and these genes were involved in various biological processes [20]. Despite the importance of LTTRs, very little information is currently available regarding their regulation of virulence factors in *A. hydrophila*.

To identify the factors responsible for the hemolytic activity of *A. hydrophila*, in this study, we individually screened for reduced hemolytic ability in a Tn5-derived library of mutants that were previously generated by our group [21]. Interestingly, we identified a LysR-type transcriptional regulator (LahS) in *A. hydrophila* NJ-35. Our study showed that, in addition to hemolytic activity, LahS positively regulates a variety of virulence factors, including motility, biofilm formation, and protease activity, and it is involved in the anti-bacterial competition ability and virulence of this bacterium. This is the first study to evaluate the role of LahS in virulence and environmental adaptation in *A. hydrophila*, the results of which are of great importance in gaining a better understanding of the pathogenesis of this bacterium.

## 2. Results

### 2.1. Isolation of Transposon Mutants with Reduced Hemolytic Activity

**Figure 1:**
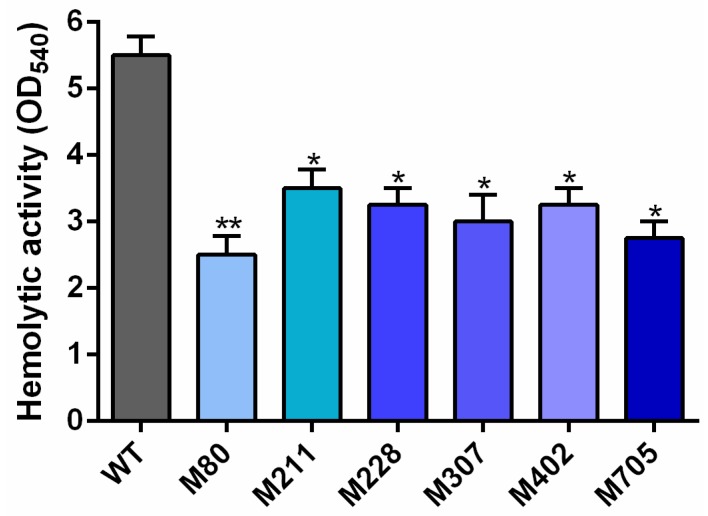
Hemolytic activity of the mutants compared to the wild-type strain. A total of six mutants were identified based on their hemolytic activities. * *p* < 0.05 or ** *p* < 0.01.

Among 947 random EZ-Tn5 transposon mutants, a total of six mutants were identified as having reduced hemolytic activity (Figure 1). The EZ-Tn5 chromosomal insertion sites of the six mutants were analyzed by Tail-PCR, followed by BLASTn searches and sequence analyses. The data revealed that the six hemolysis-associated genes that were identified in this study included those encoding for PTS alpha-glucoside transporter subunit IIBC, a LysR family transcriptional regulator, an AraC family transcriptional regulator, and three hypothetical proteins (Table 1).

### 2.2. Effect of the LysR-type Transcriptional Regulator on A. hydrophila Hemolytic Activity

**Figure 2:**
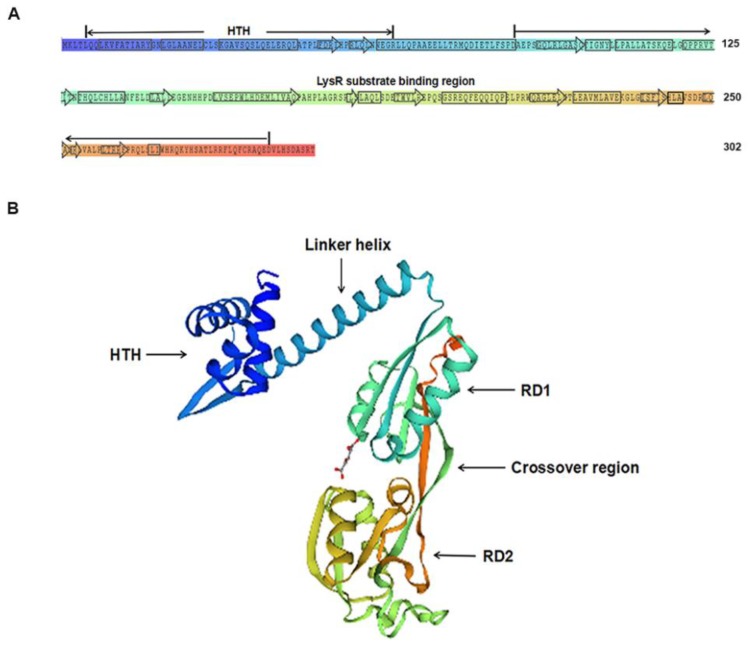
Structure of LahS protein. (**A**) Secondary structure of LysR family transcriptional regulator (LahS) protein. The N-terminal helix–turn–helix (HTH) domain and the LysR-substrate binding region were predicted using simple modular architecture research tool (SMART) software [23]. Rectangular box, α-helix; arrowheaded box, β-strand. (**B**) Predicted three-dimensional (3D) structure of LahS protein. The predicted maps were constructed with the SWISS-MODEL software [24]. Domains were shown in the corresponding position with the same colour coding, as in Figure 2A.

As shown in Figure 1, the M307 mutant exhibited decreased hemolytic activity. A sequence analysis of this strain revealed that the EZ-Tn5-disrupted in this strain encodes a protein belonging to LTTRs. This newly identified gene was designated as *lahS*. The open reading frame (ORF) of *lahS* is 909 bp, which encodes a hypothetical 34 kDa protein consisting of 302 amino acid (aa) residues. Secondary structure analysis showed that LahS protein has a conserved N-terminal DNA-binding helix–turn–helix (HTH) motif, which is located at amino acids 5–64 from N terminus (Figure 2A). In addition, there is a LysR substrate binding region that is located at amino acids 87–293, which has the ability to affect the binding capability of this protein. The two domains are connected via a long flexible linker helix that is involved in the oligomerization of the proteins (Figure 2B). The predicted three-dimensional (3D) structure of LahS showed that there are two α/β regulatory domains (RD1 and RD2) in the LysR substrate binding region, which are connected by two crossover regions (Figure 2B). 

**Figure 3:**
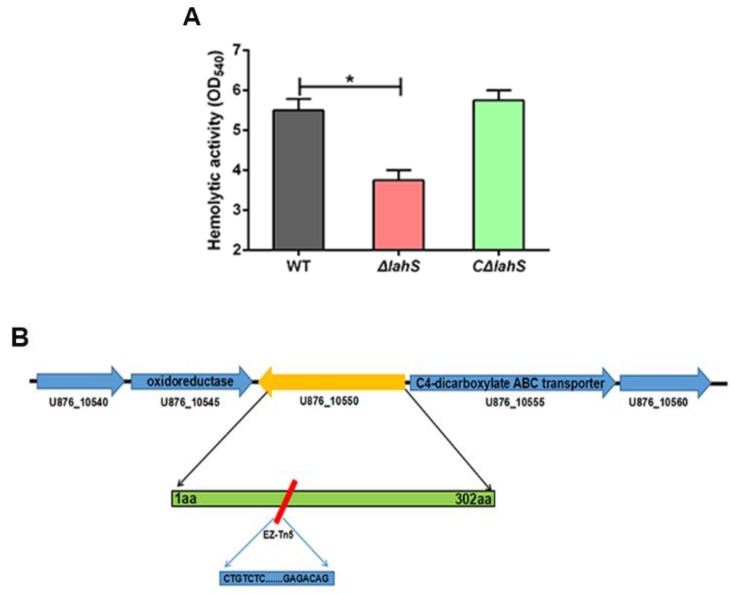
The *lahS* gene was involved in hemolytic activity of *A. hydrophila.* (**A**) Hemolytic activity of the wild-type and *lahS* mutant strains. *A. hydrophila* strains were grown overnight in Luria Bertani broth (LB) medium at 28 °C, after which 100 µL of supernatants were added to 1% sheep blood for 1 h at 37 °C. Hemolytic activity was expressed as the values measured at OD_540_. * *p* < 0.05. (**B**) Schematic diagram of the EZ-Tn5 transposon insertion in the *lahS* gene. The gene cluster shows the location of the *lahS* gene in the NJ-35 genome. The red rectangle shows the transposon insertion site in *lahS*.

To verify whether the hemolytic phenotype of the M307 mutant was due to *lahS* inactivation, we constructed *lahS* mutant and complemented strains via homologous recombination. Similar to the M307 mutant, the *lahS* deletion mutant showed decreased hemolytic activity, and the hemolytic activity in the complemented strain *CΔlahS* was restored to wild-type levels (Figure 3A). Through a BLAST analysis, *lahS* homologs were identified within the genomes of several *Aeromonas* species, including *Aeromonas salmonicida* strain A527 (89%), *Aeromonas veronii* strain TH0426 (83%), *Aeromonas caviae* strain 8LM (83%), and *Aeromonas schubertii* strain WL1483 (72%). Additionally, the transcription levels of both the upstream and the downstream genes, which encode an oxidoreductase and C4-dicarboxylate ABC transporter, respectively (Figure 3B), showed no significant differences between the *ΔlahS* and wild-type strains (Supplementary Figure S1). This finding indicated that the *lahS* mutation had no polar effect on the transcription of adjacent genes. Collectively, our findings indicate that LahS is a newly identified regulator of hemolytic activity in *A. hydrophila* NJ-35, and homologs of this regulator are present in a number of *Aeromonas* species.

### 2.3. LahS Is Involved in Biofilm Formation of A. hydrophila NJ-35

**Figure 4:**
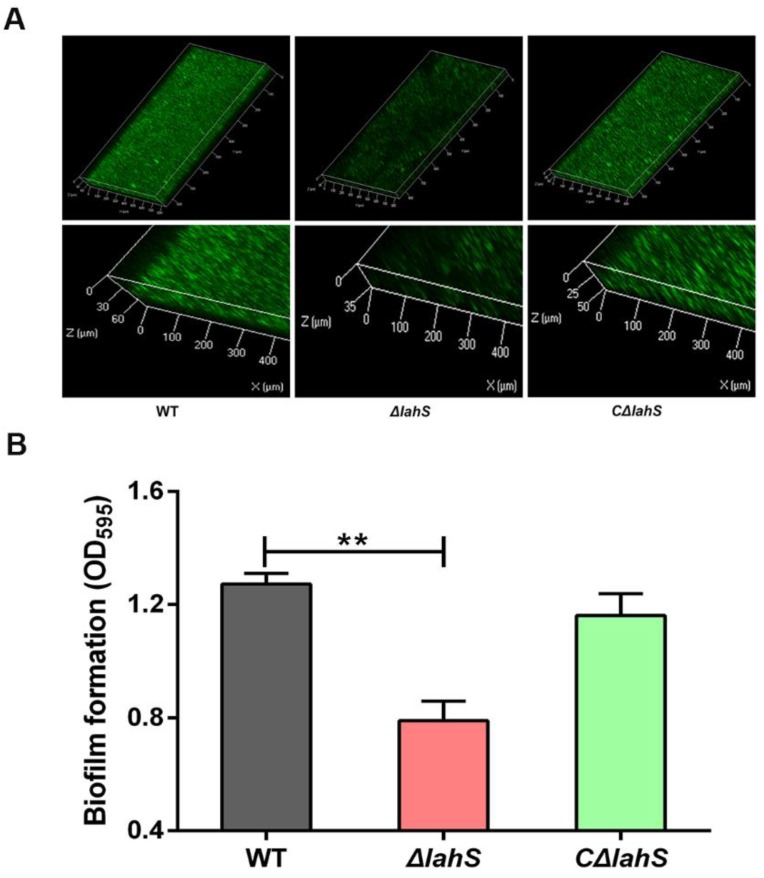
Biofilm formation of the wild-type and *lahS* mutant strains. (**A**) CLSM images of biofilms of the wild-type and *lahS* mutant strains. The viable cells exhibit green fluorescence. (**B**) Biofilm formation was measured by crystal violet staining using 96-well plates and was expressed as the values measured at OD_595_. The data are presented as the means ± SEM from three independent experiments. ** *p* < 0.01.

CLSM analysis of the *A. hydrophila* NJ-35 wild-type, *ΔlahS,* and *CΔlahS* strains showed that wild-type strain and *CΔlahS* exhibited abundant biofilm formation, while the biofilm formation phenotype of the *ΔlahS* mutant is poor when compared to that of the wild-type strain (Figure 4A). This is consistent with our crystal violet staining results at 24 h. The ability of *ΔlahS* mutant to form biofilms was significantly decreased (by 38.8%) as compared to the wild-type strain (*p <* 0.01), whereas the biofilm formation was restored to the wild-type level in the *CΔlahS* strain (Figure 4B). These data indicate that the transcriptional regulator LahS may directly or indirectly regulate biofilm formation of *A. hydrophila.*

### 2.4. LahS Influences Motility in A. hydrophila NJ-35

**Figure 5:**
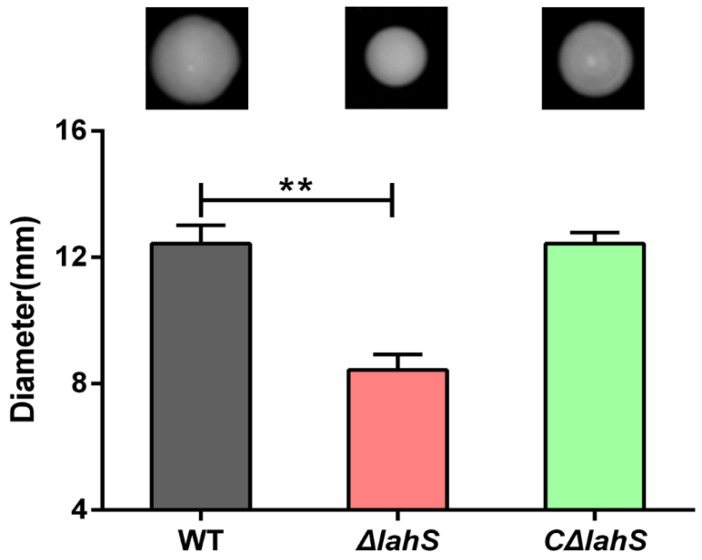
Motility of the wild-type and *lahS* mutant strains. Swimming ability was observed after culturing strains at 28 °C for 48 h on 0.3% Luria Bertani broth (LB) agar plates. The migration diameters were measured to assess the motility. The results are presented as the means ± SEM from three independent replicates. ** *p* < 0.01.

The swimming motility of *A. hydrophila* was measured by examining the distance cells migrated from the center to the periphery of the plate. As shown in Figure 5, migration diameters of 12.4 ± 0.6 and 8.4 ± 0.5 mm were measured for the wild-type and *ΔlahS* mutant strains, respectively, indicating that the swimming motility of the *ΔlahS* mutant was significantly decreased when compared with that of the wild-type strain. The motility phenotype was partially restored in the *CΔlahS* strain. This finding indicates that LahS contributes to the motility of *A. hydrophila* NJ-35.

### 2.5. LahS Contributes to the Antibacterial Activity of A. hydrophila NJ-35

**Figure 6:**
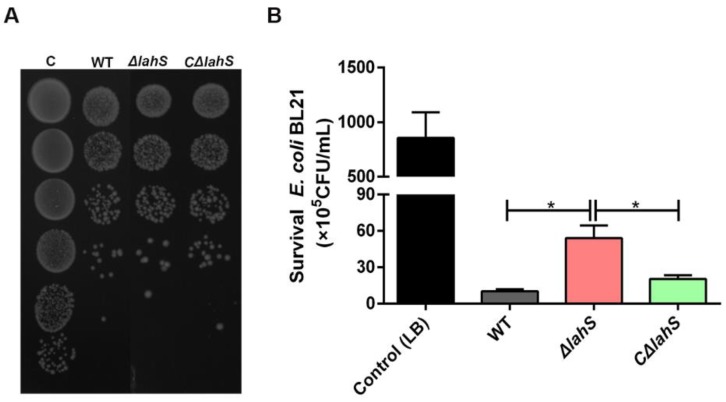
Competition ability of the wild-type and *lahS* mutant strains. (**A**) The image of competition ability is shown on Luria Bertani broth (LB) agar plate. (**B**) Quantification analysis of competition ability of the wild-type and *lahS* mutant strains. The respective bacterial strains were cultured together at a ratio of 1:1. The competition capability of *A. hydrophila* strains against *E. coli* BL21 was defined as the amount of observed *E. coli* survival after antagonism. Data are presented as the means ± SEM of three independent experiments. * *p* < 0.05.

To determine whether the transcriptional regulator LahS influences the antibacterial ability of *A. hydrophila* NJ-35, we carried out growth competition experiments by co-culturing *A. hydrophila* strains with *E. coli* BL21. The *A. hydrophila* strains were ampicillin resistant, and the *E. coli* BL21 strain contained the plasmid pET-28a (+), which confers kanamycin resistance to allow for the selection of viable *E. coli* BL21 cells. As shown in Figure 6, co-culturing of *E. coli* BL21 with *ΔlahS* mutant showed about a six-fold rise in colony-forming unit (CFU) when compared to *E. coli* that was co-cultured with the wild-type strain (*p* < 0.05). The antibacterial capacity of the *ΔlahS* mutant was restored after complementing with *lahS* gene. 

### 2.6. LahS Plays a Role in the Resistance of A. hydrophila NJ-35 to Oxidative Stress

**Figure 7:**
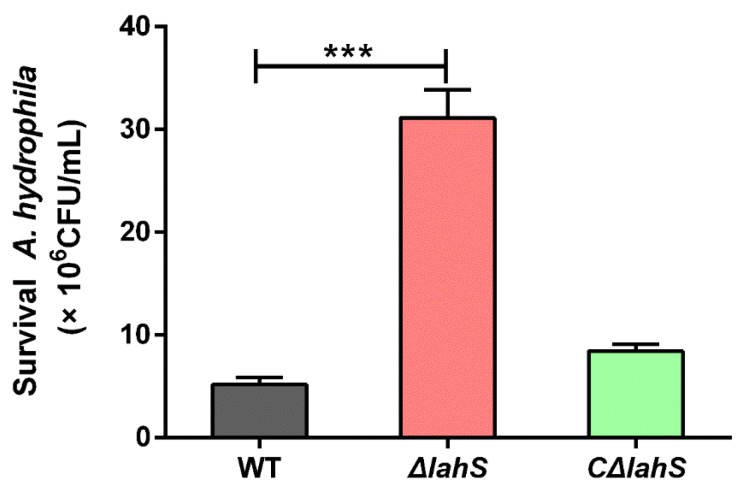
Oxidative stress resistance of the wild-type and *lahS* mutant strains. The effect of hydrogen peroxide on cell viability was examined to investigate the role of *lahS* in the resistance of *A. hydrophila* to oxidative stress. The H_2_O_2_ resistance levels were expressed as the colony-forming unit (CFU) of the viable *A. hydrophila* after treatment with H_2_O_2_. Data are presented as the means ± SEM of three independent experiments. *** *p* < 0.001.

The antioxidant abilities of *A. hydrophila* strains were determined by treating each strain with H_2_O_2_. As shown in Figure 7, the *ΔlahS* mutant was hyposensitive to H_2_O_2_ as compared with the wild-type strain (*p* < 0.001). The viable cell number of the *ΔlahS* mutant was six-fold higher than that of the wild-type strain. This anti-oxidation ability was partially restored in the *CΔlahS* strain. This result suggested that disruption of *lahS* enhanced the antioxidant capacity of *A. hydrophila* NJ-35.

### 2.7. Deletion of lahS Reduced the Protease Activity of A. hydrophila NJ-35

**Figure 8:**
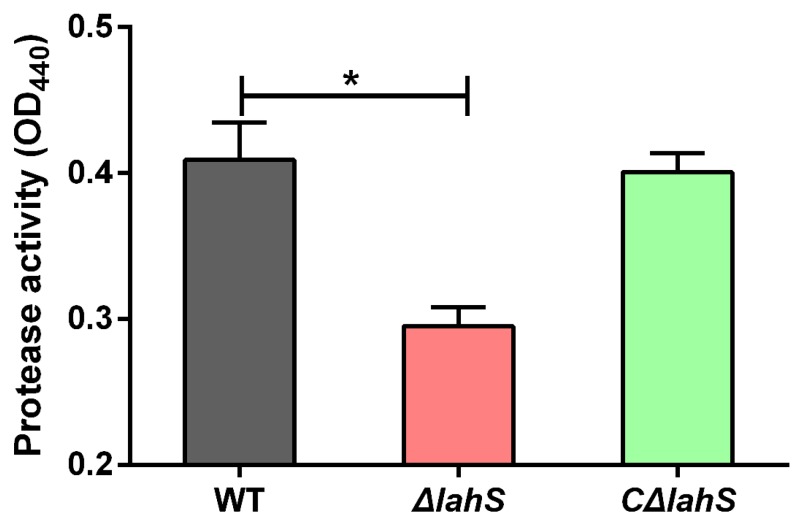
Protease activity of the wild-type and *lahS* mutant strains. The protease activity was detected using azocasein as a protease substrate and was measured at OD_440_. Data are presented as the means ± SEM of three independent experiments. * *p* < 0.05.

The culture supernatants of *A. hydrophila* strains were used to test for the presence of protease activity. The results in Figure 8 show that the *ΔlahS* mutant was significantly reduced for the production of protease (0.295 ± 0.013) as compared with that observed in the wild-type strain (0.409 ± 0.025) (*p* < 0.05). The protease activity was partially restored in the complemented strain *CΔlahS.* The result suggested that the *lahS* gene was involved in the protease activity of *A. hydrophila* NJ-35.

### 2.8. LahS Is Essential for the Virulence of A. hydrophila in Zebrafish

**Figure 9:**
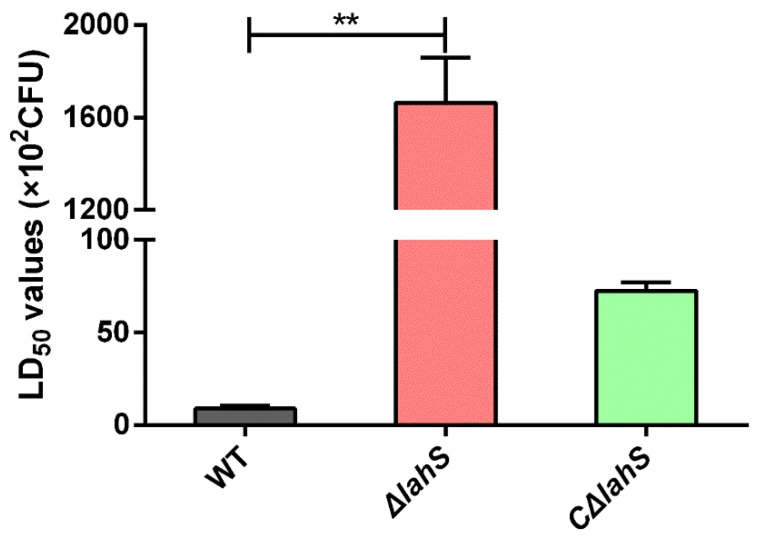
LD_50_ values of the wild-type and *lahS* mutant strains in zebrafish. The zebrafish were intraperitoneally (*i.p.*) injected with 10-fold serially diluted bacterial suspensions. The control group was *i.p.* injected with sterile phosphate buffered saline (PBS) only. The results are presented as the means ± SEM from three independent replicates. ** *p* < 0.01.

To determine whether the *lahS* gene affected bacterial virulence, the LD_50_ values of the wild type and *lahS* mutant strains were compared while using zebrafish. The LD_50_ of *A. hydrophila* NJ-35 was 8.84 × 10^2^ CFU, and all the fish were dead within three days. However, the *lahS* mutant had a LD_50_ of more than 10^5^ CFU (Figure 9). Most of the dying fish showed typical clinical signs of hemorrhagic septicemia. Colonies of *A. hydrophila* were recovered from all dead fish, and no evident external lesions were observed in the surviving fish.

### 2.9. Comparative Proteomic Analysis 

When considering that LahS functions as a transcriptional regulator, we investigated the differentially expressed profiles of the wild-type and the *lahS* mutant strains while using a label-free mass spectrometry method. The comparative proteomic analysis showed that a total of 2051 proteins matched to the Universal Protein Resource (UniProt). Thirty-four proteins were differentially expressed (change of > 1.5-fold) between the *lahS* mutant and wild type strain, including 10 upregulated and 24 downregulated proteins. A complete list of the names or locus tags of the 34 proteins is shown in Table 2. 

**Figure 10:**
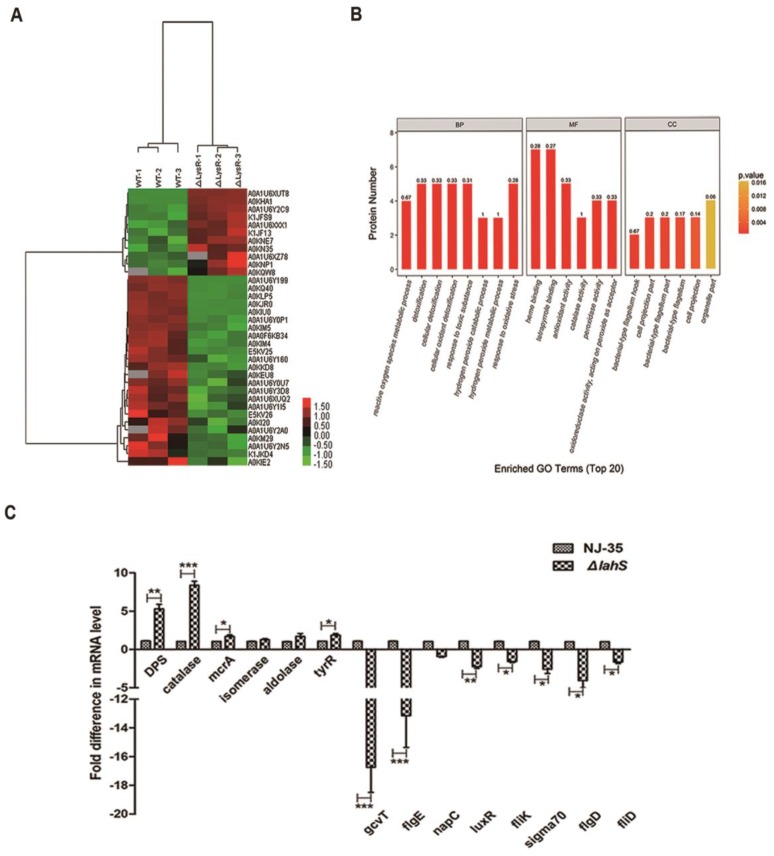
Label-free quantitative proteomics analysis of differentially expressed proteins between the wild-type and *lahS* mutant strains. (**A**) Heat map of the 34 identified proteins. Up- and down-regulated proteins are indicated in shades of green (increased) and red (decreased), respectively. (**B**) Gene Ontology (GO) classification of differentially expressed proteins. The differentially expressed proteins are grouped into three hierarchically structured terms: biological process, cellular component, and molecular function. (**C**) Relative mRNA expression levels of 14 genes coding the differentially expressed proteins in *lahS* mutant and wild-type strains. The results were expressed as n-fold increases with respect to the control. Data are presented as the means ± SEM from three independent experiments. * *p* < 0.05, ** *p* < 0.01 or *** *p* < 0.001.

To identify the relationship between the 34 different proteins, a hierarchical clustering method based on Pearson’s correlation of variances was applied while using R studio. Figure 10A shows the hierarchical clustering of the 34 identified proteins, where an increasing color intensity indicates increasing protein expression levels. To further understand the functions of these identified proteins, we classified 34 differential proteins by Gene Ontology (GO) categories, including cellular component (CC), molecular function (MF), and biological process (BP) (Figure 10B). According to the analysis of CCs, the majority of the proteins were involved in cell projection and flagellum. According to the analysis of BPs, the primary functions were cellular detoxification and antioxygenation. According to the analysis of MFs, the assayed proteins regulated by LahS could be classified into six categories, as follows: heme binding, tetrapyrole binding, antioxidant activity, catalase activity, peroxidase activity, and oxidoreductase activity. In addition, to determine the accuracy of the mass spectrometry results, 14 of these 34 genes (six of the upregulated genes and eight of the downregulated genes) were randomly selected for further verification by qRT-PCR, the results of which showed that the quantitative PCR results were consistent with the proteomic data (Figure 10C).

## 3. Discussion

Hemolytic molecules are the major contributors to the hemorrhagic septicemia that is characteristic of *A. hydrophila* infections in fish, although this bacterium uses a variety of virulence factors [5]. However, prior to this study, little was known regarding the regulation of these hemolytic factors in this bacterium. In the present study, we identified a novel hemolysis-associated regulator (LahS) in *A. hydrophila* that belongs to the LTTRs. Interestingly, we observed that the LahS participated in different biological activities in this pathogen, including motility, biofilm formation, environmental adaptability, and virulence.

To investigate the global impact of the *lahS* deletion on protein expression in *A. hydrophila* NJ-35, we performed a label-free quantification analysis between the *ΔlahS* and wild-type strains. From this analysis, it was observed that LahS regulated the expression of a series of proteins that were involved in a wide range of biological processes, including oxidative stress, transcriptional regulation, DNA replication, and metabolism. LTTRs have been reported to regulate motility in many bacteria, but the molecular mechanisms are different in different strains. For example, LeuO in *V. cholerae* O1 modulates motility by cooperating with the nucleoid-associated protein H-NS to repress *vieSAB* transcription [25]. In *E. coli*, LrhA controls the transcription of flagellar, motility, and chemotaxis genes by regulating the expression of the master regulator FlhDC [26]. In this study, 34 proteins were observed to be differentially expressed in the *lahS* mutant as compared to the wild-type strain. Among the downregulated proteins, four proteins are involved in flagellin formation, including FlgE, FliK, FlgD, and FliD. In addition, the expression of ORF (U876_07335), which is 94% identical to FlrA from *A. hydrophila* AH-3 by a BLASTp analysis, was downregulated in the *lahS* mutant. FlrABC is known as the polar flagellum master regulator of a four-step hierarchical for the expression of flagellar genes in *A. hydrophila* [27]. Therefore, we speculate that LahS may control motility by positively regulating the expression of FlrABC.

Bacterial biofilm formation has been divided into three stages: the planktonic stage, the monolayer stage and the biofilm stage [28]. The transition between different stages is initiated by various environmental signals through the action of specific transcription factors. In *V. cholerae*, LeuO regulates the transition from the monolayer to the biofilm stage by the modulation of exopolysaccharide VPS gene transcription [29]. In addition to stage-specific regulatory functions, certain members of LysR family have roles in biofilm formation through a combination of direct and indirect regulation. For example, OxyR from *H. parasuis* regulates the DNA-binding transcriptional regulator FabR, which is directly involved in biofilm formation, as well as some membrane-related genes that are indirectly related to biofilm formation, such as an outer membrane assembly protein and an outer-membrane lipoprotein carrier protein [20]. In the present study, no proteins that are directly related to biofilm formation were identified among the differentially expressed proteins between the *ΔlahS* and wild-type strains, but RNA polymerase sigma 70 (σ^70^), which has been reported to participate in the biofilm formation process [30], was observed to be downregulated. This led to us to speculate that deletion of *lahS* might downregulate the expression of the σ^70^ gene, which in turn, leads to a decrease in biofilm formation. In addition, a previous study confirmed that flagellar genes were actively transcribed during the planktonic stage, and motility and chemotaxis were important for the initiation of bacterial biofilm formation [31]. Therefore, there is another possibility that the reduction in motility may affect biofilm accumulation.

It is particularly noteworthy that we observed that the inactivation of *lahS* resulted in a significant downregulation of a LuxR family transcriptional regulator. LuxR regulators are key players in quorum sensing (QS), which coordinates the expression of a variety of genes, including those encoding virulence factors, motility, and biofilm formation [32]. Our previous study showed that the LuxR-type response regulator, AhyR, contributes to exoprotease and hemolysin production and the virulence of *A. hydrophila* [33]. In the present study, LahS was observed to be involved in motility, biofilm formation, interbacterial competition, hemolytic and proteolytic activities and virulence in zebrafish. All of these findings led us to speculate that the diverse role exhibited by LahS in *A. hydrophila* virulence might be achieved by upregulating the expression of a LuxR regulator. This speculation is further strengthened by a recent study that was conducted by Gao et al. [34], which indicated that a LysR-type transcriptional regulator (VqsA) is an important QS regulator in *Vibrio alginolyticus* and it plays essential roles in QS-regulated phenotypes, such as type VI secretion system 2 (T6SS2)-dependent interbacterial competition, biofilm formation, exotoxin production, and in vivo virulence. In this regard, it will be interesting to elucidate whether a correlation between LahS and QS exists in *A. hydrophila*.

Members of the LysR family have been widely reported to participate in regulating the antioxidant capacity and environmental adaptability, of which OxyR is the most thoroughly studied [35,36,37]. OxyR controls oxidative stress by acting as an activator [38] or a repressor [39] for defensive factors, such as catalase. In this study, the inactivation of *lahS* resulted in a substantial increase in the expression level of the catalases (U876_01465), which might partly explain the phenomenon that antioxidant capacity of the *lahS*-deleted strain was significantly higher than that of the wild-type strain.

In conclusion, our data suggest that the hemolysis-associated regulator LahS is positively involved in motility, biofilm formation, protease production, anti-bacterial competition ability, and virulence, but it negatively regulates the antioxidant capacity in *A. hydrophila*. The comparative proteomic analysis revealed that LahS directly or indirectly controls the expression of 34 proteins involved in flagellar assembly, cellular metabolism, oxidative stress, and environmental adaptability. It will be interesting to further examine the roles of LahS in virulence expression in *A. hydrophila* and to explore whether interfering with its function is an effective way to defend against this bacterial infection.

## 4. Materials and Methods 

### 4.1. Strains, Plasmids and Growth Conditions

The bacterial strains and plasmids that were used in this study are listed in Table 3. *A. hydrophila* NJ-35, which belongs to the ST251 clonal group, was isolated from diseased cultured crucian carp in the Jiangsu province of China in 2010 [40]. The genome sequence of *A. hydrophila* NJ-35 has been published in GenBank (accession number CP006870). *A. hydrophila* and *E. coli* were cultured in Luria Bertani broth (LB) at 28 and 37 °C, respectively. When necessary, chloramphenicol (Cm) (Sigma Louis, MO, USA), kanamycin (Kan) (Sigma), or ampicillin (Amp) (Sigma) were added to the medium. 

### 4.2. Screening Transposon Insertion Mutants for Hemolytic Activity

A library containing 947 random EZ-Tn5 transposon mutants based on *A. hydrophila* NJ-35 was previously constructed in our laboratory [21]. All the mutants were assayed for hemolytic activity, as described previously [44], with some minor modifications. Briefly, *A. hydrophila* strains were grown overnight in LB medium at 28 °C, diluted to an optical density of 0.2 at 600 nm (OD_600_), and pelleted by centrifugation at 10,000× *g* for 10 min. Next, the supernatants were filter-sterilized while using 0.22-µm (pore-size) membrane filters, and 100 µL of supernatant was double ratio diluted and dispensed into 96-well polystyrene plates. Subsequently, 100 µL of 1% sheep blood was added to each well and mixed completely. The plates were incubated for 1 h at 37 °C without agitation, followed by an overnight incubation at 4 °C. The plates were centrifuged at 1000× *g* for 10 min, after which 100-µL aliquots of supernatants were transferred into a new 96-well polystyrene plate. The OD_540_ was monitored while using a spectrophotometer (BIO-RAD, Hercules, CA, USA). 

### 4.3. Identification of Insertion Sites by Tail-PCR

The insertion sites of the EZ-Tn5 in the chromosome of *A. hydrophila* NJ-35 were identified by Tail-PCR. The primers that were used in this study are listed in Supplementary Table S1. Six specific primer pairs (SP1–SP6) were designed to amplify the DNA sequence from either end of the transposon. Degenerate primers AD1 to AD6 were paired with SP1 to SP6, respectively, to amplify the flanking sequences of the inserted EZ-Tn5. The detailed operation of the Tail-PCR was performed, as previously described [21]. After Tail-PCR, the specific products were gel purified and sequenced. The BLASTn program was used to compare the DNA sequence with the genome of the reference strain NJ-35 to confirm the sequences flanking the EZ-Tn5.

### 4.4. Construction of a lahS Mutant and Complemented Strains

The *lahS* deletion mutant strain was constructed, as previously described [42]. Briefly, two flanking regions of the target gene were amplified and ligated by PCR using two sets of primer pairs, *lahS-1/lahS-2* and *lahS-3/lahS-4* (Table 4). Next, the fusion fragment was generated with the primer pair *lahS-1/lahS-4* and then was inserted into pYAK1 and transformed into *E. coli* SM10 [41]. The recombination vector from the donor *E. coli* SM10 strain was conjugated into the recipient *A. hydrophila* NJ-35 strain. LB agar plates containing 100 µg/mL Amp and 34 µg/mL Cm were used to select for isolates in which the plasmid had integrated into the chromosome via recombination. The positive colonies were inoculated into LB broth supplemented with 20% sucrose to induce a second crossover event, and aliquots of this culture were subsequently spread onto LB agar plates containing 20% sucrose to generate the deletion of mutants. The *lahS* mutant was confirmed by sequencing the deleted region and flanking DNA in the mutated strain.

The complemented *ΔlahS* strain was constructed using the shuttle plasmid pMMB207 [43]. The complete *lahS* gene and its putative promoter and terminator regions were amplified using the primer pair *lahS*-C-F/R and then ligated into the pMMB207 vector. The recombinant plasmid pMMB207-*lahS* was transformed into the *ΔlahS* mutant strain by bacterial conjugation to generate the complemented strain *CΔlahS*, which was verified by PCR. 

### 4.5. Biofilm Formation Assay 

To compare the biofilm formation ability of strains NJ-35 and *ΔlahS*, confocal laser scanning microscopy (CLSM) was performed to analyze the three-dimensional architecture of biofilms as previously described [45]. Briefly, the stationary phase bacterial cultures were adjusted to an OD_600_ of 1.0 and then diluted 1:1000 in LB medium. Two milliliters of these dilutions were subsequently added into each well of 6-well plate containing pre-sterilized microscopic glass slides for biofilm growth. After incubating at 28 °C for 24 h to allow for biofilm development, the glass slides were carefully washed with phosphate buffered saline (PBS) to remove the planktonic cells. Next, 20 μL of fluorescein diacetate (FDA, Sigma, Louis, MO, USA) was added to each glass slide, which were then protected from light for 20 min. The slides were then observed by CLSM (Carl Zeiss LSM700, Oberkochen, Germany) to observe the biofilms using an argon laser. Seven random spots were measured for each of the three replicate glass slides. The image stacks were recorded under identical conditions (i.e., similar area and vertical resolution). Sterilized glass slides incubated with fresh LB medium were used as a control group.

Biofilm formation was also quantitatively measured by crystal violet staining, as previously described [46]. *A. hydrophila* strains were grown to an OD_600_ of 0.6–0.8 in LB broth at 28 °C and then diluted to an OD_600_ of 0.1. Next, 200-µL aliquots of suspensions (1:100 dilution in fresh LB) were dispensed into 96-well polystyrene plates, which were incubated at 28 °C for 24 h without shaking. The medium was decanted, and the wells were washed three times with sterile PBS. Next, the bacterial cells were fixed with 200 µL of methanol for 15 min, after which they were air dried at room temperature. After drying, 200 μL of a crystal violet solution (1% *w/v*) was added to each well and the cells were stained for 10 min. The wells were then rinsed with ddH_2_O to remove the unbound crystal violet. The bound crystal violet was solubilized using 95% ethanol for 10 min, and the optical density was measured at OD_595_. The assay was performed in three independent experiments.

### 4.6. Motility Assay

The swimming motility assay was performed using 0.3% agar plates, as previously described [47]. *A. hydrophila* strains were grown to the log phase in LB broth at 28 °C, and 1 μL of each culture (5 × 10^8^ CFU/mL bacteria) was stabbed into motility assay agar plates. The plates were incubated at 28 °C for 48 h, after which motility was assessed by measuring the migration diameter of the bacterial cells. The assay was performed in three independent experiments.

### 4.7. Anti-Bacterial Competition Assay

The *E. coli* inhibition assay was performed as previously described with some modifications [48]. *A. hydrophila* and *E. coli* BL21 strains grown to an OD_600_ of 1.0 and then were concentrated 10 times. Cells were mixed at a ratio of 1:1, spotted onto 0.22-µm sterile filters on LB plates, and incubated at 28 °C for 3 h. *E. coli* BL21 cells that were mixed with equal volume of LB media was used as a control. Then, the spots were serially diluted in 1 mL LB medium, and the survival *E. coli* was quantified by serial dilution in LB and visualized on LB plates containing kanamycin. The assay was performed in three replicates.

### 4.8. Oxidative Stress Resistance Test

For oxidative stress tests, 4 mM H_2_O_2_ was freshly prepared before each experiment and was filter-sterilized using 0.22-µm (pore-size) membrane filters. Log-phase cultures were normalized to an OD_600_ of 0.5. Next, 400-µL aliquots were added to 100 µL H_2_O_2_ and the mixtures were incubated at 28 °C for 1 h. The oxidation was terminated with 2000 U of catalase for 10 min. The number of viable *A. hydrophila* was counted via serial dilution of the suspensions and plating on LB agar plates. The assay was performed in three independent experiments.

### 4.9. Protease Activity

*A. hydrophila* strains were grown overnight in LB medium at 28 °C and the OD_600_ values were normalized to 2.0 with fresh LB medium. Next, the cells were pelleted by centrifugation at 10,000× *g* for 10 min, and the supernatants were then filter-sterilized using 0.22-µm (pore-size) membrane filters. Subsequently, 250-µL aliquots of supernatants were added to 250 µL of 0.5% (*w/v*) azocasein in 50 mM Tris-HCl (pH 8.0) and then incubated at 37 °C for 2 h. After incubating, 500 µL of 10% (*w/v*) trichloroacetic acid (TCA) was added and the mixture was incubated on ice for 30 min. The cells were then pelleted by centrifugation at 10,000× *g* for 10 min, and 500 µL of the supernatants were added to 500 µL NaOH. The azodye was measured at OD_440_.

### 4.10. Determination of LD_50_ in Zebrafish

The animal experiment was carried out in accordance with the animal welfare standards and guidelines of the Animal Welfare Council of China and was approved by the Ethical Committee for Animal Experiments of Nanjing Agricultural University, China [permit number: SYXK (SU).2017-0007]. Zebrafish were supplied by the Pearl River Fishery Research Institute, Chinese Academic of Fishery Science. The animal-challenge experiment was performed according to a previous study [49]. Log-phase bacteria were washed three times with sterile PBS and the suspensions were serially tenfold diluted from 5 × 10^2^ to 5 × 10^7^ CFU/mL. Ten zebrafish per group were intraperitoneally (*i.p.*) injected with 20 µL of the suspensions in PBS. An additional 10 zebrafish were injected with 20 µL of sterile PBS as the negative control. Mortality was recorded for seven days, and the LD_50_ values were calculated by the method of Reed and Muench [50].

### 4.11. Comparative Proteomic Analysis 

*A. hydrophila* strains were grown overnight in LB broth at 28 °C, after which the bacterial cells were washed three times with PBS and then resuspended in lysis buffer. The bacterial suspensions were then ruptured by sonication at 4 °C and centrifuged (12,000× *g*, 30 min, 4 °C) to collect the supernatants. Next, 10% (*w/v*) TCA was added to the supernatants, which were incubated in ice-cold water for 30 min. The supernatants were centrifuged at 12,000× *g* for 10 min and then washed three times with ice-cold acetone. The proteins were harvested by centrifugation at 12,000× *g* for 10 min and analyzed using a label free mass spectrometry method. For quantitative proteomics analysis, 200 μg samples were digested with trypsin (1:50 *w/w*; Sigma Louis, MO, USA) at 37 °C for 24 h. Peptide mixtures were fractionated by nano-liquid chromatography and analyzed by mass spectrometry (MS). MS data were searched against uniprot_Aeromonas_hydrophila_27500_20170605.fasta (27,500 total entries, downloaded 5 June 2017). The identified proteins were analyzed by GO categories, KEGG enrichment, and clustering analyses. 

### 4.12. Quantitative Reverse Transcription-PCR (qRT-PCR)

To validate the proteomic results, we used qRT-PCR to measure the transcription levels of randomly selected genes. The primer pairs used in this assay are shown in Supplementary Table S2. Total RNA was isolated using an E.Z.N.A. bacterial RNA isolation kit (Omega, Beijing, China). cDNA synthesis was performed while using a PrimeScript RT reagent kit (TaKaRa, Dalian, China). qRT-PCR was performed to quantify each target transcript using a QuantiTect SYBR green PCR kit (Qiagen, Valencia, CA, USA). The constitutively expressed *recA* gene was chosen as a reference gene for qRT-PCR, and the 2^−ΔΔ*C*^_t_ method was used as previously described [51].

### 4.13. Statistical Analysis

Statistical analyses were performed while using GraphPad Prism 6. Student’s *t* test was used for examining the differences between the wild-type and mutant strains. *p* values of <0.05 were considered significant.

## Figures and Tables

**Table 1:** Characteristics of the hemolysis-reduced mutants screened by transposon mutant library.

Mutant	Locus Tag	Insertion Site/Gene Length (bp) ^a^	Function ^b^
M80	U876_21575	2/480	hypothetical protein
M211	U876_23300	125/1587	PTS alpha-glucoside transporter subunit IIBC
M228	U876_22535	102/246	hypothetical protein
M307	U876_10550	440/909	LysR family transcriptional regulator
M402	U876_02250	330/516	hypothetical protein
M705	U876_01475	711/857	Arac family transcriptional regulator

^a^ Insertion sites were identified using Tail-PCR and sequence analysis. ^b^ Putative functions were obtained from the NCBI BLAST online server [22].

**Table 2:** Differentially expressed proteins in *lahS* mutant compared to the wild type strain.

Locus Tag	Predicted Function	Fold Change
**Up-regulated proteins**		
U876_17600	Hypothetical protein	28.34991961
U876_01465	Catalase	11.31448355
U876_16540	Peroxidase	2.09906885
U876_04415	Cytochrome C biogenesis protein CcsA	2.043010753
U876_04895	Triose-phosphate isomerase	2.939215876
U876_07195	Fructose-6-phosphate aldolase	2.67060636
U876_05460	Penicillin-sensitive transpeptidase	6.074663579
U876_13305	Transcriptional regulator	2.236916529
U876_10585	Prolyl-tRNA synthetase	2.143335753
U876_23535	Chromosome partitioning protein ParA	2.1254663
**Down-regulated proteins**		
U876_07270	Flagellar hook protein FlgE	0.123192009
U876_16245	Flagellar hook-length control protein FliK	0.396639518
U876_07265	Flagellar hook capping protein FlgD	0.446402163
U876_14260	Flagellar hook-associated protein FliD	0.49944046
U876_14545	Phospho-2-dehydro-3-deoxyheptonate aldolase	0.021640145
U876_09970	Phospho-2-dehydro-3-deoxyheptonate aldolasee	0.067411226
U876_11610	LuxR family transcriptional regulator	0.279023788
U876_07335	Fis family transcriptional regulator	0.366104356
U876_14125	Glycine cleavage system protein T	0.014741915
U876_08310	Hypothetical protein	0.22058777
U876_14865	Cytochrome C	0.243506128
U876_17595	Hydroxylamine reductase	0.267712471
U876_13425	Thioredoxin reductase	0.280916369
U876_14870	Nitrate reductase	0.304735753
U876_22700	YeeE/YedE family protein	0.381751269
U876_14250	Hypothetical protein	0.401170621
U876_07930	Cytochrome c biogenesis protein CcmH	0.426902415
U876_15300	Hypothetical protein	0.43795805
U876_13000	Methionine gamma-lyase	0.448173659
U876_01200	Cytochrome C	0.45592439
U876_16185	RNA polymerase sigma 70	0.457438622
U876_16300	Peptidase C80	0.481165879
U876_01650	Single-stranded DNA-binding protein	0.487195726
U876_09765	Electron transporter HydN	0.494528201

**Table 3:** Bacterial strains and plasmids used in this study.

Strain or Plasmid	Description ^a^	Source or Reference
**Strains**		
NJ-35	Wild-type, isolated from diseased crucian carp, in China	Collected in our laboratory
SM10	*E. coli* strain, λ*pir*^+^, Kan^r^	[41]
BL21	*E. coli* strain, *F*^−^, *ompT, hsdS (rB^−^mB^−^), gal, dcm* (DE3)	Invitrogen
*ΔlahS*	*lahS* deletion mutant from NJ-35	This study
*CΔlahS*	*ΔlahS* complemented with pMMB-*lahS*	This study
**Plasmid**		
pET 28a (+)	Kan^r^, F1 origin	Novagen
pYAK1	R6K-ori suicide vector, SacB^+^, Cm^r^	[42]
pYAK1-*lahS*	pYAK1 carrying the flanking sequence of *lahS*, Cm^r^	This study
pMMB207	Low-copy-number vector, Cm^r^	[43]
pMMB-*lahS*	Plasmid pMMB207 carrying the complete ORF of *lahS*	This study

^a^ Characteristics of strains or plasmids. Kan^r^, kanamycin resistant; Cm^r^, chloramphenicol resistant.

**Table 4:** Primers used for construction of *lahS* mutant and complemented strain.

Primer	Sequence (5′–3′)
*lahS-1*	CAGGTCGACTCTAGAGGATCCTGGAGGCAATGAAGGTGA
*lahS-2*	ATATCCAAACGCCAATGGGATGATCGG
*lahS-3*	TCCCATTGGCGTTTGGATATCCTGAACGATT
*lahS-4*	GAGCTCGGTACCCGGGGATCC CGCTCACGGTCAGGTAAC
*lahS*-C-F	GAGCTCGGTACCCGGGGATCCATGAAGCTGACCCTGCAAC
*lahS*-C-R	CAGGTCGACTCTAGAGGATCCTCAGGTGCGGCTGGC

## Supplementary Materials

Supplementary materials can be found at http://www.mdpi.com/1422-0067/19/9/2709/s1. Figure S1: Fold difference in mRNA level; Table S1: Primers used for Tail-PCR; Table S2: Primers used for qRT-PCR.
