# Supplementary material for: Discovery of lahS as a Global Regulator of Environmental Adaptation and Virulence in Aeromonas hydrophila
Source: Int J Mol Sci. 2018 Sep 11;19(9):2709. doi: 10.3390/ijms19092709 (PMC6163582; doi:10.3390/ijms19092709)
Supplement: Supplementary file 1 [file ijms-19-02709-s001.zip › Supplementary Files/Table S1 S2.doc]

***T****able S1 Primers used for Tail-PCR.*

| Primer | Sequence (5′–3′) |
| --- | --- |
| SP1 | TTGGTTGTAACACTGGCAGAGCATT |
| SP2 | CGGGACGGCGGCTTTGTTGAATAA |
| SP3 | CCAACTGGTCCACCTACAACAAAGC |
| SP4 | TGCTGCTGGCTACCCTGTGGAA |
| SP5 | TCGGGTGCGGAGAAAGAGGTAA |
| SP6 | CAGGTTGAACTGCTGATCTTCGGAT |
| AD1 | (G/C)TTGNTA(G/C)TNCTNTGC |
| AD2 | NTCGA(G/C)T(A/T)T(G/C)G(A/T)GTT |
| AD3 | (A/T)GTGNAG(A/T)ANCANAGA |
| AD4 | NGTCGA(G/C)(A/T)GANA(A/T)GAA |
| AD5 | TG(A/T)GNAG(G/C)ANCA(G/C)AGA |
| AD6 | AG(A/T)GNAG(A/T)ANCA(A/T)AGG |

***T****able S2 Primers used for qRT-PCR.*

| Primer | Sequence (5′–3′) |
| --- | --- |
| *DPS*-RT-S | TCCGATTGGTCTTGATACCGT |
| *DPS*-RT-A | TGCAGCTCGAAGAACTGGTTG |
| *catalase*-RT-S | TCTGGTTGATGGAAAAGTTGG |
| *catalase*-RT-A | GATGTCATGGGTCACGGTAAA |
| *mcrA*-RT-S | GATCACCTTGAATGCCCCTTA |
| *mcrA*-RT-A | TGGTATAGACGTGCAGACCCA |
| *isomerase*-RT-S | ACAGAGGATGGGAATCAGACC |
| *isomerase*-RT-A | AATGTGAGCCAGCACAGACAG |
| *aldolase*-RT-S | TAATGGACGGATTGGTGGTAA |
| *aldolase*-RT-A | ATCTCGATACTGCCGATGTGG |
| *tyrR*-RT-S | GGCAAAGCGGGAGACGAA |
| *tyrR*-RT-A | ACGAGCAGGAGGTGAAGGTC |
| *gcvT* -RT-S | CCATCAACTACGGCTCACAGC |
| *gcvT* -RT-A | GGTAGAAGGTCTCGCCAAGGT |
| *flgE*-RT-S | GGGCACCTTGGGTAGCAT |
| *flgE*-RT-A | TTCCAGCGAACGGGAGTT |
| *napC*-RT-S | CTCTGGGTGCTGCTGTTGATG |
| *napC*-RT-A | GGTTGGCGTAGTGGATGGTGT |
| *luxR*-RT-S | GCAACGCCAGTTCAAAGA |
| *luxR*-RT-A | GATGGTGAGCGGATAGAGG |
| *fliK*-RT-S | CAACCTACCGATAACAATGGC |
| *fliK*-RT-A | ATTCTCACCCTGTTCGCTTAA |
| *flgD*-RT-S | AGGTGACCAGAAGGGCAACA |
| *flgD*-RT-A | ACCCAGGATCACGCTGTCG |
| *sigma 70*-RT-S | ATGGCTTCGGCGATGGTG |
| *sigma 70*-RT-A | CAGGCAGGTATGATTGGATTG |
| *fliD*-RT-S | CAATGGCGATAACTTCGGTCT |
| *fliD*-RT-A | CCATCAACAACAATCTCAGCACT |
| *recA* -RT-S | CGACCCCATCTATGCCGC |
| *recA* -RT-A | CCATCTCACCTTCGATTTCCG |
